# Impact of Bundle Branch Block on Permanent Pacemaker Implantation after Transcatheter Aortic Valve Implantation: A Meta-Analysis

**DOI:** 10.3390/jcm10122719

**Published:** 2021-06-19

**Authors:** Justine M. Ravaux, Michele Di Mauro, Kevin Vernooy, Silvia Mariani, Daniele Ronco, Jorik Simons, Arnoud W. Van’t Hof, Leo Veenstra, Suzanne Kats, Jos G. Maessen, Roberto Lorusso

**Affiliations:** 1 Department of Cardio-Thoracic Surgery, Heart and Vascular Centre, Maastricht University Medical Centre (MUMC), 6202 AZ Maastricht, The Netherlands; mdimauro1973@gmail.com (M.D.M.); s.mariani1985@gmail.com (S.M.); daniele.ronco@LIVE.IT (D.R.); Jorik_Simons@hotmail.com (J.S.); suzanne.kats@mumc.nl (S.K.); j.g.maessen@mumc.nl (J.G.M.); roberto.lorussobs@gmail.com (R.L.); 2 Department of Cardiology, Maastricht University Medical Centre (MUMC), 6202 AZ Maastricht, The Netherlands; kevin.vernooy@mumc.nl (K.V.); arnoud.vant.hof@mumc.nl (A.W.V.H.); l.veenstra@mumc.nl (L.V.); 3 Cardiovascular Research Institute Maastricht (CARIM), Maastricht University Medical Center, 6202 AZ Maastricht, The Netherlands; 4 Department of Cardiology, Radboud University Medical Center (Radboudumc), 6525 GA Nijmegen, The Netherlands; 5 Department of Medicine and Surgery, Circolo Hospital, University of Insubria, 21100 Varese, VA, Italy

**Keywords:** transcatheter aortic valve implantation, right bundle branch block, left bundle branch block, permanent pacemaker implantation

## Abstract

Data regarding the impact of infra-Hisian conduction disturbances leading to permanent pacemaker implantation (PPI) after transcatheter aortic valve implantation (TAVI) remain limited. The aim of this study was to determine the impact of right and/or left bundle branch block (RBBB/LBBB) on post-TAVI PPI. We performed a systematic literature review to identify studies reporting on RBBB and/or LBBB status and post-TAVI PPI. Study design, patient characteristics, and the presence of branch block were analyzed. Odds ratios (ORs) with 95% CI were extracted. The final analysis included 36 studies, reporting about 55,851 patients. Data on LBBB were extracted from 33 studies. Among 51,026 patients included, 5503 showed pre-implant LBBB (11.9% (10.4%–13.8%)). The influence of LBBB on post-TAVI PPI was not significant OR 1.1474 (0.9025; 1.4588), *p* = 0.2618. Data on RBBB were extracted from 28 studies. Among 46,663 patients included, 31,603 showed pre-implant RBBB (9.2% (7.3%–11.6%)). The influence of RBBB on post-TAVI PPI was significant OR 4.8581 (4.1571; 5.6775), *p* < 0.0001. From this meta-analysis, the presence of RBBB increased the risk for post-TAVI PPI, independent of age or LVEF, while this finding was not confirmed for patients experimenting with LBBB. This result emphasizes the need for pre-operative evaluation strategies in patient selection for TAVI.

## 1. Introduction

Atrio-ventricular conduction disturbances and subsequent permanent pacemaker implantation (PPI) represent frequent complication after transcatheter aortic valve implantation (TAVI) [1,2]. Notably, infra-Hisian conductions’ desynchrony such as left bundle branch block (LBBB) and right bundle branch block (RBBB) remain an ongoing issue in TAVI [3,4], especially considering TAVI as well-established therapeutic approach for patients with aortic stenosis at high surgical risk [5], while considerable advances in procedural techniques tend to extend TAVI indications to patients with a lower surgical risk [6]. Mechanical stress on the aortic valve annulus, deterioration of the ventricular septum, and local edema may all injury the atrio-ventricular conduction during the TAVI procedures [1,3]. In patients with pre-existing conduction system impairments, such additional procedural-related factors may contribute to a higher post-TAVI PPI rate. Consequently, complications such as PPI thus remain a substantial barrier to extending this technique to operable patients who would otherwise undergo surgery [7]. Therefore, better patient selection and identification of pre-operative risk factors for progression of conduction disturbances, and subsequently PPI, are decisive [8]. Current data about the clinical impact of bundle branch block on post-TAVI PPI remain controversial [9,10]. Left bundle branch block (LBBB) occurs in 5 to 65% in TAVI patients and leads to PPI in 15 of 20% of cases [11]. Pre-operative right bundle branch block (RBBB) is present in 10 to 21% of patients and results in up to 40% of post-operative PPI, making pre-operative RBBB the most important patient-related factor [2,12]. However, the prognostic value on pre-existing infra-Hisian conduction disturbances on post-TAVI PPI remains unclear [10,11,12].

We aim to investigate the clinical impact of pre-operative RBBB/LBBB on PPI after TAVI.

## 2. Materials and Methods

### 2.1. Research Strategy

This meta-analysis was performed in accordance with the Preferred Reporting Items for Systematic Reviews and Meta-Analyses (PRISMA) and the research strategy was developed according to available guidance from the Cochrane Collaboration. A broad, computerized literature search was performed to identify all relevant studies from Embase, Cochrane database, and PubMed exploring Medical Subject Heading (MeSH) terms related to pre-operative RBBB or pre-operative/new-onset LBBB in TAVI population. The PubMed database was searched entering the following key words: “Pacemaker, Artificial” [Mesh] OR pacemaker implantation AND “Transcatheter Aortic Valve Replacement” [Mesh] OR transcatheter aortic valve replacement AND “Bundle-Branch Block” [Mesh]. We restricted the research to English publications. Last access to the database was on 1 November 2020. The search was limited to studies in human.

### 2.2. Eligibility Criteria and Studies Selection

Studies were included in the final analyses if patients were >18 years (I); >250 patients were included in the main analysis, in order to provide data interpretation of the most consistent clinical series (II); and studies provided a description of pacemaker status of the population (III). Furthermore, articles with no possible extraction of the presence of RBBB/LBBB were excluded. Pre-operative RBBB/LBBB and new-onset RBBB/LBBB were both included in the present analysis. Systematic review and meta-analyses were not taken in account. Studies describing cardiac surgery procedures were also left out. The selected articles underwent extensive evaluation at title and abstract level by two independent researchers (J.R. and M.D.M.) to assess the potential inclusion in the meta-analysis. Discrepancies were solved by consensus with the intervention of a third reviewer (R.L.). There were no duplicate data.

### 2.3. Statistical Analysis

Calculation of an overall proportion from studies reporting a single proportion was performed using a meta-analytic approach by means of metaprop function of meta package in R. A logit-transformation was performed as suggested by Warton & Hui [13] to calculate confidence intervals (CIs) for individual study results. A Clopper–Pearson approach and a DerSimonian–Laird estimator were used to estimate the between-study variance [14]. Total proportion with 95% Cl was reported. Funnel plot and Egger’s test were used for estimation of publication bias. The primary endpoint was 30-day or in-hospital PPI after TAVI, so odds ratios (OR) with 95% CI were extracted from 36 studies. Statistical pooling of OR was performed using a random effect model with 95% CI. Forest plots were used to plot the effect size, either for each study or overall. We calculated the I2 statistics (0%~100%) to explain the between-study heterogeneity, with I2 ≤ 25% suggesting more homogeneity, 25% < I2 ≤ 75% suggesting moderate heterogeneity, and I2 > 75% suggesting high heterogeneity [15,16]. If the null hypothesis was rejected, a random effects model was used to calculate pooled effect estimations. If the null hypothesis was not rejected, a fixed effects model was used to calculate pooled effect estimations [14]; 95% CI was also reported. Forest plots were used to plot the effect size, either for each study or overall. Publication bias was evaluated by graphical inspection of funnel plot; estimation of publication bias was quantified by means of Egger’s linear regression test [17]. In the case of moderate or high heterogeneity, influence analysis was performed with different approaches: Baujat plot [18] and a leave-one-out sensitivity analysis were performed by iteratively removing one study at a time to confirm that our findings were not driven by any single study. Meta-regression analysis was performed, reporting results as regression coefficient (i.e., Beta) and *p*-value. One removed analysis was performed as a sensitivity analysis. “Meta package” in R-studio version 1.1.463 (2009–2018) was used. Because this study was a systematic review and meta-analysis based on published articles, ethical approval was waived by the institutional review board of the University Hospital of Maastricht.

## 3. Results

### 3.1. Study Inclusion

Our search yielded 877 records initially screened at the title and abstract level, with 222 papers fully reviewed for eligibility. There were no duplicate data. Ultimately, 36 studies were identified and provided data for the research analysis (Supplementary Figure S1).

### 3.2. Baseline Characteristics of Included Patients and Permanent Pacemaker Implantation Details

Table 1 shows the baseline characteristics of the included studies. The 36 studies included a total of 55,851 patients in the final analysis, from 2005 to 2018 [19,20,21,22,23,24,25,26,27,28,29,30,31,32,33,34,35,36,37,38,39,40,41,42,43,44,45,46,47,48,49,50,51,52,53,54]. The mean age of the patients was 81.9 years, with a mean STS score of 8.3. Only five studies were prospective in nature [35,40,41,44,51]. The PPI details in the included studies are reported in Table 2. The overall incidence of PPI reached 15.2%, ranging from 4.3% to 32%.

### 3.3. Influence of LBBB on PPI

Data on LBBB were extracted from 33 studies [19,20,21,22,23,24,25,26,27,28,29,30,31,32,33,34,35,36,37,38,39,40,41,42,43,47,48,49,50,51,52,53,54]; among the 51,026 patients included in the analysis, there were 5503 showing pre-implant LBBB. The cumulative proportion of LBBB was 11.9% (10.4%–13.8%) (Supplementary Figure S2). Heterogeneity was high (I^2^ 96.4% [95.6%; 97.0%]). No publication bias was found (*p* = 0.2921). The cumulative proportion of LBBB in a subset of 7315 patients with post-TAVI PPI was 13.0% (10.6%–15.8%) with high heterogeneity (I^2^ 87.7% [83.7%; 90.6%]) and no publication bias (*p* = 0.3856) (Supplementary Figure S3). The cumulative proportion of LBBB in a subset of 43,650 patients without post-TAVI PPI was 12.3% (10.5%–14.4%) with high heterogeneity (I^2^ 96.5% [95.8%; 97.1%]) and no publication bias (*p* = 0.6200) (Supplementary Figure S4).

**Figure 1:**
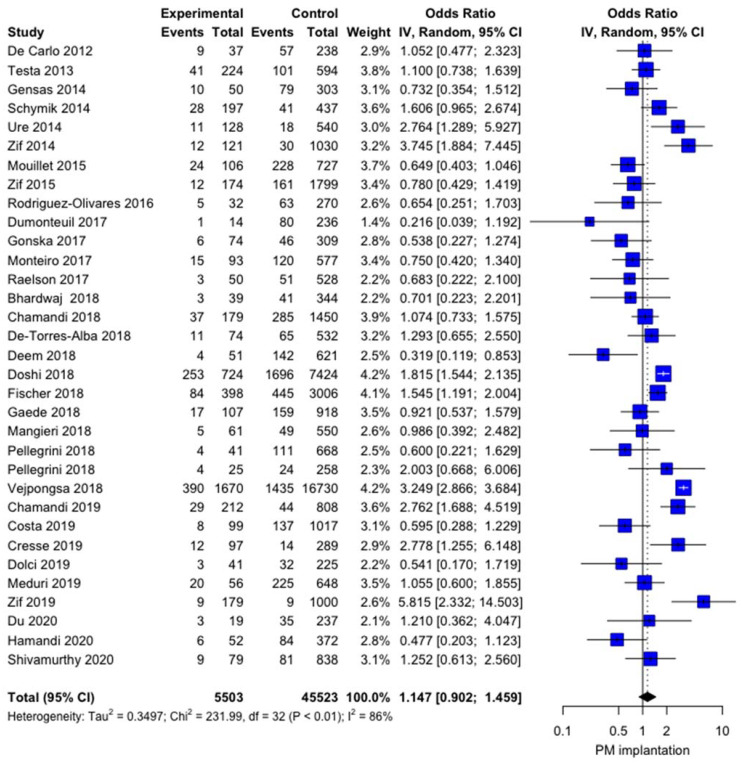
Forest plot comparing the effect of LBBB on the rate of post-TAVI PPI. IV= interval variable; 95% CI = confidence interval.

**Figure 2:**
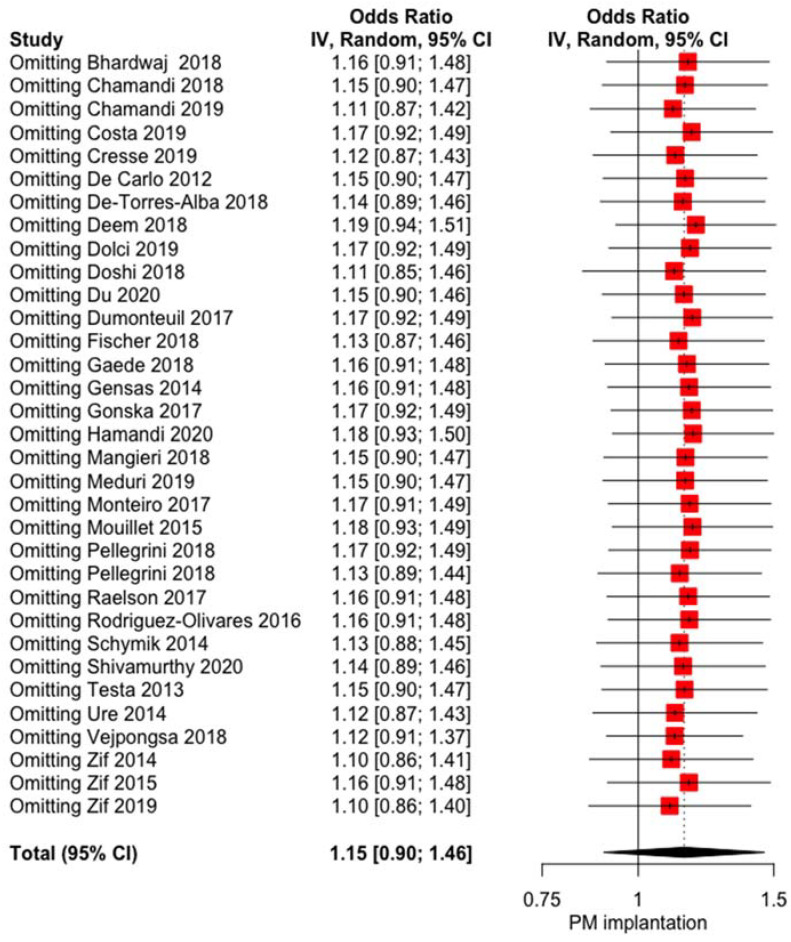
Forest plot leave one-out analysis comparing the effect of the presence of LBBB on the rate of post-TAVI PPI. IV= interval variable; 95% CI= confidence interval.

The influence of LBBB on post-TAVI PPI was not significant OR 1.1474 (0.9025; 1.4588, *p* = 0.2618) with high heterogeneity I^2^ = 86.2% [81.7%; 89.6%] and no publication bias (*p* = 0.7100) (Figure 1). The baujat plot (Supplementary Figure S5) shows that the study by Vejpongsa et al. [37] may impact high heterogeneity, even if the sensitivity analysis does not confirm this hypothesis, as no influence of LBBB on post-TAVI PPI rate was evidenced at the leave-one out analysis (Figure 2). Meta-regression failed to identify some modifiers: age (r = −0.0592, *p* = 0.4292), left ventricular ejection fraction (LVEF, r = 0.0754, *p* = 0.1741), and year of the study (r = −0.0008, *p* = 0.9893) did not show any influence on the meta-analytic results (Supplementary Figures S6–S8).

### 3.4. Influence of RBBB on PPI

Data on RBBB were extracted from 28 studies (19–46); among the 46,663 patients included in the analysis, there were 31,603 showing pre-implant RBBB. The cumulative proportion of RBBB was 9.2% (7.3%–11.6%) (Supplementary Figure S9). Heterogeneity was high (97.8% [97.3%; 98.1%]). No publication bias was found (*p* = 0.1112). The cumulative proportion of RBBB in a subset of 6932 patients with post-TAVI PPI was 24.7% (19.6%–30.6%) with high heterogeneity (94.6% [93.2%; 95.8%]) and no publication bias (*p* = 0.1023) (Supplementary Figure S10). The cumulative proportion of RBBB in a subset of 39,670 patients without post-TAVI PPI was 6.3% (4.9%–8.1%) with high heterogeneity (I^2^ 96.6% [95.9%; 97.3%]) and no publication bias (*p* = 0.2659) (Supplementary Figure S11).

**Figure 3:**
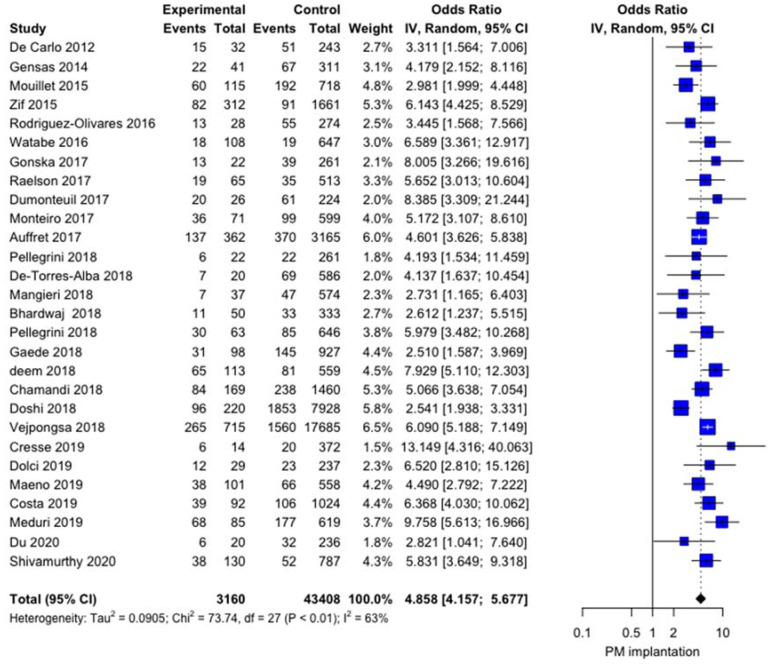
Forest plot comparing the effect of RBBB on the rate of post-TAVI PPI.

**Figure 4:**
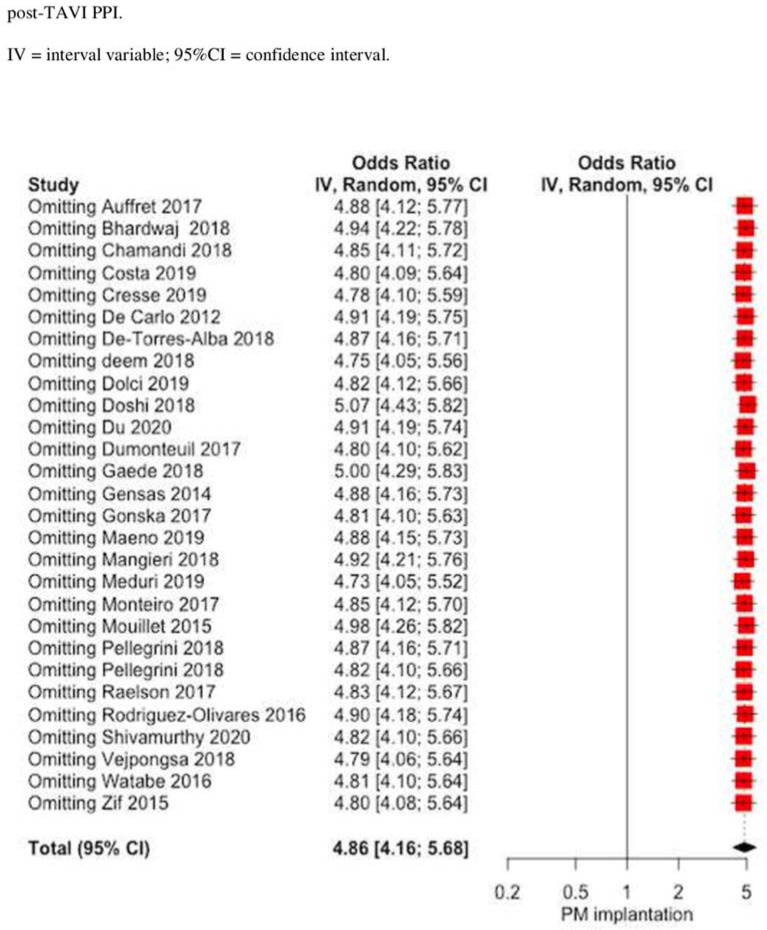
Forest plot leave one-out analysis comparing the effect of the presence of RBBB on the rate of post-TAVI PPI.

The influence of RBBB on post-TAVI PPI was significant, with OR 4.8581 (4.1571; 5.6775), *p* < 0.0001 with moderate heterogeneity I^2^ = 63.4% [45.1%; 75.6%] and no publication bias (*p* = 0.937) (Figure 3). The baujat plot (Supplementary Figure S12) shows that two studies [36,37] may impact the heterogeneity. Sensitivity analysis confirms the impact of RBBB on the post-TAVI PPI rate even at the leave-one out analysis (Figure 4). Meta-regression failed to identify some modifiers: age (r = −0.0592; *p* = 0.4292), left ventricular ejection fraction (LVEF, r = 0.0246, *p* = 0.3755), and year of the study (r = 0.0489, *p* = 0.3038) did not show any influence on meta-analytic results (Supplementary Figures S13–S15).

## 4. Discussion

Our meta-analysis demonstrates the impact of bundle branch block on post-TAVI PPI. Our study is derived from 36 studies reporting clinical outcomes in 55,851 patients receiving TAVI and presenting bundle branch conduction disturbances. The main results of this study can be assumed as follows: (i) the presence of LBBB does not influence post-TAVI PPI; (ii) the presence of RBBB has a significant impact on post-TAVI PPI.

The prevalence of LBBB in TAVI candidates is closed to 10% according to previous studies [45,55,56] and new-onset LBBB occurs in ≅30% after TAVI, depending on the type of prosthesis [9,57]. In the present study, the cumulative proportion of LBBB was 11.9%, including pre-operative LBBB and new-onset LBBB after TAVI. This rate is thus in accordance with previously published data [4,58]. The impact of LBBB on post-TAVI PPI remains controversial [49,50,59]. Data from the PARTNER experience [50,53] suggested that new-onset LBBB increases the rate of post-TAVI PPI. Moreover, an analysis at a national registry level as the prospective open TAVI Karlsruhe registry [48] identified slightly more PPI in patients with persistent new-onset LBBB. However, current data do not promote prophylactic PPI in patients presenting new-onset LBBB after TAVI [60] and some studies failed to identify LBBB as predictors for post-TAVI PPI [58,61]. In our study, the influence of LBBB on post-TAVI PPI was not significant. These controversial results can be related to various definitions of LBBB according to the degree of QRS prolongation defining the LBBB [58,61,62,63] or to the high heterogeneity among the current studies [64]. Moreover, only three studies [22,50,53] quantified LBBB as left anterior fascicular or left posterior fascicular block, which may underestimate the impact of LBBB on post-TAVI PPI. As development of high-degree atrio-ventricular block is shown to be a common complication in patients with pre-existing LBBB (or RBBB) after TAVI [65], we shared the view of Waksman and colleagues [66] emphasizing the need for routine electrophysiology evaluation in patients undergoing TAVI in order to limit post-TAVI PPI. Electrophysiology testing differentiates the supra-nodal conduction disturbances from the infra-nodal ones by analyzing the atrial-His and the His-ventricular intervals. By integrating these values into the baseline electrocardiogram, we may be able to predict the evolution of the atrio-ventricular conduction in high-grade atrio-ventricular block. Such data may help the clinicians to choose between an aggressive strategy of early post-TAVI PPI or, on the contrary, an expectative strategy, avoiding some unnecessary post-TAVI PPI. The results of current ongoing trials investigating the use of electrophysiology-based algorithmic approaches with HV measurements as a guide for PPI, particularly in patients with LBBB (Clinical Monitoring Strategy vs. EP-Guided Algorithmic in LBBB Patients Post-TAVR (NCT03303612); The MARE Study (NCT02153307); Prospective Validation of a Pre-Specified algorithm for the management of conduction disturbances following transcatheter aortic valve replacement PROMOTE (NCT04139616)), are expected with impatience.

Pre-existing RBBB has been demonstrated in 6.9% [67] up to 13.6% [44] of patients undergoing TAVI and is a common underlying conduction disturbance in TAVI patients [10]. In this study, the cumulative proportion of RBBB among the included studies reached 9.2%, also in accordance with the studies of Auffret and colleagues [45], reporting a prevalence of RBBB of 10.3% in TAVI candidates. Several studies have already highlighted the role of pre-existing RBBB in the development of atrioventricular conduction, leading to post-TAVI PPI [2,3,22,26,33,39,41]. Our findings confirm the impact of such a condition on post-TAVI PPI, without the influence of age or LVEF. As previous studies emphasized the higher all-cause mortality and the poorer clinical outcomes in patients with pre-existing RBBB [10,45], bradyarrhythmia events may participate in the poorer outcomes of these patients. However, this analysis does not provide the cause of such discrepancies between RBBB and LBBB. The fragility of the right bundle branch during minor trauma or procedures may partially explain its highest incidence in the general population and, consequently, the more important impact of RBBB in post-TAVI PPI [68]. We look forward to having definitive data and guidelines to determine the optimal management and monitoring of patients with RBBB undergoing TAVI [60].

The current guidelines with respect to post-TAVI PPI [69] do not specify the adequate timing for post-TAVI PPI. Previously, the 2013 European Society Guidelines [70] recommended a clinical rhythm observation period of 7 days in the presence of high-grade trio-ventricular block before proceeding to post-TAVI PPI. In the current meta-analysis, the range of timing for PPI varied from day 0 to 1 year, also emphasizing the need for PPI after discharge and the influence of atrio-ventricular conduction in the long term [49]. This hypothetical observation period may be shortened or lengthened according to patient-related factors and electrical findings. Once again, electrophysiological study may identify some high-risk patients for the development of high-grade atrio-ventricular disturbances and, thereby, adjust the observation rhythm period in such patients [71].

As the definitive impact of infra-Hisian conduction disturbances on post-TAVI PPI has not definitively been clarified yet, specific recommendations for the type of valves used in such patients must be done with caution. However, some studies investigated the impact of the valves used on post-TAVI conduction disturbances [3,44,49,61]. Indeed, Franzoni and colleagues [61] found a higher incidence of LBBB when using a Medtronic CoreRevalving System with respect to the Edwards Sapien Valve. A more intra-ventricular implantation of the stent valve frame may result in higher rate of post-TAVI PPI [49]. Furthermore, the gap between the lower edge of the coronary cusp and the end of the frame of the prosthetics valve has been shown to be greater in patients with new-onset LBBB than in patients with post-operative conduction disturbances [61], emphasizing the role of the prosthesis placement in the development of post-operative conduction disturbances. Nevertheless, the requirement for post-TAVI PPI is multifactorial and studies incorporating electrophysiological findings with procedural data are mandatory [72].

### Study Limitations

This meta-analysis has several limitations to be acknowledged. First, this meta-analysis possesses all the inherent bias associated with this investigation technique. Second, including pre-operative LBBB and new-onset LBBB in the analysis may be considered as a limitation for the interpretation of the final results, as the pre-operative LBBB can be related to other cardiovascular conditions and the new-onset LBBB can be related to procedural/interventional aspects. However, as our primary outcome was 30-day or in-hospital PPI, we can afford to compare both LBBB as conduction disturbances present after TAVI, which can lead to PPI, independently of the chronic or acute apparition of the LBBB. Third, distinction of LBBB in left anterior fascicular or left posterior fascicular block was lacking in the majority of the included studies, thereby not providing enough information to perform in-depth analysis. Further research in this respect is currently needed. Finally, analysis of individual patient-level data may provide further understandings.

## 5. Conclusions

This meta-analysis found that patients with the presence of RBBB have higher risk for post-TAVI PPI, with no influence of age or LVEF. This finding has not been confirmed for patients experimenting with LBBB. Pre-operative evaluation strategies, including electrophysiological characteristics, are crucial in further extending patient selection for TAVI.

## Figures and Tables

**Table 1:** Baseline characteristics of included studies (n = 36). TAVI, transcatheter aortic valve implantation.

Study	Year	Study Design (Nr Centers)	Sample Size	Age (Years)	STS-Score (%)	Inclusion Period	Valve Type	Follow-Up (Months) *	Approach for TAVI	Mortality at 30 Days
De Carlo et al. [19]	2012	Retrospective (3)	275	82.4	na	Sep 2007–Jul 20120	100% MCV	12	na	3%
Gensas et al. [20]	2014	Retrospective (18)	353	82	14.4	Jan 2008–Feb 2012	85.8% SE 14.2% BE	60	na	na
Mouillet et al. [21]	2015	Retrospective (29)	833	82	14.1	Jan 2010–Oct 2011	100% SE	8	na	9.3%
Nazif et al. [22]	2015	Retrospective (21)	1973	84.5	11.4	May 2007–Sept 2011	100% BE	12	na	6.6%
Rodriguez-Olivares et al. [23]	2016	Retrospective (1)	302	81	na	Nov 2005–Jan 2015	67.2% SE 21.2% BE 11.6% ME	na	na	na
Gonska et al. [24]	2017	Retrospective (1)	283	79.9	6.7	na	100% ES3	na	na	na
Raelson et al. [25]	2017	Retrospective (1)	578	85,5	Na	Mar 2009–Dec 2014	21% SE 79% BE	1	na	na
Dumonteil et al. [26]	2017	Retrospective (14)	250	84	6,3	Oct 2012–May 2014	100% ME	12	100% TF	4%
Monteiro et al. [27]	2017	Retrospective (22)	670	81.8	10.7	Jan 2008–Jan 2015	74% MCV 26% ES	na	96% TF 4% others	na
Pellegrini et al. [28]	2018	Retrospective (3)	283	80.8	6	Jan 2014–Jan 2016	100% SE	12	100% TF	na
De-Torres-Alba et al. [29]	2018	Retrospective (1)	606	81.6	na	Jan 2014–Jan 2017	100% BE	na	na	na
Mangieri et al. [30]	2018	Retrospective (1)	611	84.4	6.9	Oct 2007–Jul 2015	51.7% BE 33.7% SE	12	na	na
Bhardwaj et al. [31]	2018	Retrospective (1)	383	83	9	Jan 2012–July 2016	82% BE 18% SE	9	84% TF	na
Pellegrini et al. [32]	2018	Retrospective (3)	709	81	na	Jan 2014–Jan 2016	100% BE	na	100% TF	1.6%
Gaede et al. [33]	2018	Retrospective (1)	1025	81.9	na	2010–2015	na	2.4	na	na
Nadeem et al. [34]	2018	Retrospective (1)	672	81.4	7.4	2011–2017	na	12	na	na
Chamandi et al. [35]	2018	Prospective (9)	1629	81.5	10.9	May 2009–Feb 2015	50.3% BE 49.7% SE	48	na	42.3%
Doshi et al. [36]	2018	Retrospective (na)	8148	82.5	na	Jan 2012–Dec 2014	na	na	na	na
Vejpongsa et al. [37]	2018	Retrospective (na)	18,400	81.2	na	Jan 2012–Dec 2013	na	na	TF 75.5% 24.5% TA	na
Cresse et al. [38]	2019	Retrospective (1)	386	83	na	Apr 2008–Jun 2017	na	na	na	na
Dolci et al. [39]	2019	Retrospective (1)	266	80	na	Feb 2014–Feb 2018	100% BE	12	84% TF 16% TA	na
Costa et al. [40]	2019	Prospective (1)	1116	82	4.4	June 2007–Feb 2018	61.8% SE 27.2% BE 0.5% ME 10.5% Others	72	97% TF 3% others	3.9%
Meduri et al. [41]	2019	Prospective (1)	704	82.5	6.6	na	34% SE 66% ME	12	na	na
Du et al. [42]	2020	Retrospective (1)	256	76.5	7.1	Mar 2013–Oct 2018	na	12	Na	3.3%
Shivamurthy et al. [43]	2020	Retrospective (1)	917	80	na	Nov 2011–Feb 2017	na	na	89.7% TF 10.3% TA	na
Studies reporting only on RBBB status in pacemaker population (n = 3)										
Watanabe et al. [44]	2016	Prospective (9)	749	85	6.9	Oct 2013–Aug 2015	100% BE	16.4	78.5% TF 18.8% TA 2.7% T iliac	4%
Auffret et al. [45]	2017	Retrospective (na)	3527	82	na	na	55.8% BE 44.2% SE	23	79.6% TF 16.4% TA 1.9% T aortic 2.1% TS	7.2%
Maeno et al. [46]	2019	Retrospective (1)	659	83	na	Jan 2013–Dec 2015	85% BE 15% SE	19.1	na	2.6%
Studies reporting only on LBBB status in pacemaker population (n = 8)										
Testa et al. [47]	2013	Retrospective (na)	818	82	na	Oct 2007–Apr 2011	100% SE	9	88.6% TF 11.4% TS	5.5%
Schymik et al. [48]	2014	Retrospective (1)	624	82	na	May 2008–Apr 2012	80.8% BE 19.2% SE	12	na	na
Urena et al. [49]	2014	Retrospective (4)	668	79.5	na	na	100% BE	13	2.8% TAortic 42.9% TA 54.3% TF	na
Nazif et al. [50]	2014	Retrospective (21)	1151	84	11.2	Mar 2007–Mar 2009	100% BE	12	57% TF 43% TA	3.6%
Fischer et al. [51]	2018	Prospective (18)	3404	81.5	5.9	Feb 2005–Oct 2017	46% SE 54% BE	22	82.2% TF 2.2% TS 14.2% TA 1.4% T aortic	5.7%
Chamandi et al. [52]	2019	Retrospective (9)	1020	80.5	6.6	May 2007–Feb 2015	48% BE 52% SE	36	84% TF 11% TA 2% Taortic 3% TS	na
Nazif et al. [53]	2019	Retrospective (51)	1179	81.2	5.5	Dec 2011–Nov 2013	100% BE	24	83.5% TF 16.5% Tthoracic	1.3%
Hamandi et al. [54]	2020	Retrospective (1)	424	82	7.6	Jan 2012–Mar 2016	87% SE 13% BE	12	85% TF 13% TA 3% TAortic	1.3%

Values are n (%), mean = SD, or median (interquartile range) as appropriate. * Follow-up is reported as mean or median as given by the authors. LBBB = left bundle branch block; RBBB = right bundle branch block; SE = self-expandable; BE = balloon-expandable; ME = mechanically-expandable; TF = trans-femoral; TS = trans-subclavian; TA = trans-apical; T iliac = trans-iliac; T aortic = trans-aortic.

**Table 2:** Pacemaker-related details in included studies. PPI, pacemaker implantation.

Studies Reporting on RBBB and LBBB Status in Pacemaker Population (n = 25)									
De Carlo et al. [19]	70% AVB 3% SSS 27% others	4	24%	32	37	15	9	* lower MCV implantation below aortic annulus * RBBB * left anterior hemiblock * longer PR interval	na
Gensas et al. [20]	na	na	25.2%	41	50	22	10	* pre-existing RBBB * balloon pre-dilatation * CoreValve use	na
Mouillet et al. [21]	na	na	30.3%	115	106	60	24	na	na
Nazif et al. [22]	79% AVB 17.3% SSS	3	8.8%	312	174	82	12	* Pre-existing RBBB * Prosthesis to LV outflow tract diameter ratio * LV-end diastolic diameter	* longer duration of hospitalization * higher rates of repeat hospitalization and mortality or repeat hospitalization at 1 year
Rodriguez-Olivares et al. [23]	na	na	22.5%	28	32	13	5	na	*more LVOT oversizing associated with higher PPI
Gonska et al. [24]	94.2% AVB 3.8% B 2% others	4,3	18.4%	22	74	13	6	* baseline AV1B * preprocedural complete RBBB	na
Raelson et al. [25]	82% AVB	3	9%	65	50	19	3	na	na
Dumonteil et al. [26]	88.9% AVB 5.9% others	3	32%	26	145	20	14	* baseline RBBB * LV outflow tract overstretch >10%	* trend lower PPI rate at 30 days with shallower (<=5mm) implant depth
Monteiro et al. [27]	na	na	20.1%	71	93	36	15	* previous RBBB * mean aortic gradient >50mmHg * MCV	na
Pellegrini et al. [28]	71.5% AVB 3.5% SSS 25% B	na	10%	22	25	6	4	* higher EuroSCORE	na
De-Torres-Alba et al. [29]	96% AVB 1.4% B 2.6% others	na	12.5%	20	74	7	11	na	na
Mangieri et al. [30]	84% AVB 8.4% B	0,3	8.8%	37	61	7	5	na	na
Bhardwaj et al. [31]	na	na	11.5%	50	39	11	3	* PPI with short-term reduction in QoL without long-term implications	na
Pellegrini et al. [32]	71.3% AVB 5.2% SSS 23.5% B	na	16.2%	63	41	30	4	* increase in prosthesis oversizing	na
Gaede et al. [33]	90% AVB 8% SSS 2% B	4	14.7%	98	107	31	17	* pre-existing RBBB * CoreValve prosthesis	Predictors of lack of recovery of AVB * prior RBBB * higher mean aortic valve gradient * post-dilatation of the prosthesis
Nadeem et al. [34]	na	na	21.7%	113	51	65	4	na	* PPI more likely to have heart failure admissions * PPI trend toward increased mortality
Chamandi et al. [35]	76.7% AVB 5.6% SSS 3.1% B 14.6% others	2	19.8%	169	179	84	37	na	* PPI higher rates of rehospitalization due to heart failure and combined endpoint of mortality or heart failure rehospitalization * PPI lesser improvement in LVEF over time, particularly in patients with reduced LVEF before TAVI
Doshi et al. [36]	na	na	24%	220	724	96	253	* female sex * AF * LBBB * AVB	na
Vejpongsa et al. [37]	na	2	9.9%	715	1670	265	390	na	na
Cresse et al. [38]	na	4	6.7%	14	97	6	12	na	* RBBB, LBBB, △PR >40 ms associated with PPI
Dolci et al. [39]	80%AVB 11% B 9% others	4	13%	29	41	12	3	* baseline RBBB * QRS width immediately after TAVI	na
Costa et al. [40]	84.8% AVB 4.1%SSS 11% Others	na	13%	92	99	39	8	na	* PPI associated with increased 6 years mortality * baseline RBBB higher chance of being dependent at follow-up
Meduri et al. [41]	90% AVB 6% B 4% others	2	28.4%	85	56	68	20	* baseline RBBB * mean depth of valve implantation	* medically-treated diabetes mellitus in LOTUS valve patients
Du et al. [42]	89.5% AVB	8.7	14.8%	20	19	6	3	na	na
Shivamurthy et al. [43]	na	na	9.8%	130	79	38	9	na	na
**Studies reporting only on RBBB status in pacemaker population (n = 3)**									
Watanabe et al. [44]	na	na	4.9%	108	na	18	na	na	na
Auffret et al. [45]	na	na	16.5%	362	na	137	na	na	na
Maeno et al. [46]	77.9% AVB 11.5% SSS 10.6%	na	15.8%	101	na	38	na	na	na
**Studies reporting only on LBBB status in pacemaker population (n = 8)**									
Testa et al. [47]	na	na	17.4%	na	224	na	41	na	* LBBB associated with higher short-term PPI
Schymik et al. [48]	na	na	10.8%	na	197	na	28	* chronic AF * baseline RBBB * MCV	na
Urena et al. [49]	55.5% AVB SSS 20.7% 24.1% B	365	4.3%	na	128	na	11	na	na
Nazif et al. [50]	na	na	5.1%	na	121	na	12	na	* LBBB with higher PPI and failure of LVEF
Fischer et al. [51]	na	na	15.5%	na	398	na	84	* pre-existing LBBB increase risk of death but not late PPI	* pre-existing LBBB associated with lower pre-operative LVEF
Chamandi et al. [52]	na	na	7.2%	na	212	na	29	na	na
Nazif et al. [53]	na	na	4.8%	na	179	na	9	na	* LBBB associated with PPI and repeat hospitalizations
Hamandi et al. [54]	na	na	18%	na	52	na	6	na	na

Values are *n* (%), mean = SD, or median (interquartile range) as appropriate. * Follow-up is reported as mean or median as given by the authors. PPI = permanent pacemaker implantation; AVB = atrio-ventricular block, SSS = Sick Sinus Syndrom; B = bradycardia; AV = atrio-ventricular; LVEF = left ventricle ejection fraction; VVI = single chamber device; DDD = dual chamber device; MCV = Medtronic CoreValve; RBBB = right bundle branch block; LVF = left ventricle function; BMI = body mass index; QoL = quality of life; AF = atrial fibrillation; LBBB = left bundle branch block; TAVI = trans-catheter aortic valve implantation; na = not available.

## Data Availability

No new data were created or analyzed in this study. Data sharing is not applicable to this article.

## Supplementary Materials

The following are available online at https://www.mdpi.com/article/10.3390/jcm10122719/s1, Figure S1. Flowsheet of the included studies, Figure S2. Forest plot pooling the proportion of LBBB in 33 studies, Figure S3. Forest plot pooling the proportion of LBBB in subset of 7315 patients with post-TAVI PPI. Figure S4. Forest plot pooling the proportion of LBBB in subset of 43,650 patients without post-TAVI PPI, Figure S5. Baujat plot: impact of the studies on overall heterogeneity in studies reporting on LBBB status, Figure S6. Bubble plots: influence of age on risk for post-TAVI PPI in patients with LBBB, Figure S7. Bubble plots: influence of LVEF on risk for post-TAVI PPI in patients with LBBB, Figure S8. Bubble plots: influence of the year of the study on risk for post-TAVI PPI in patients with LBBB, Figure S9. Forest plot pooling the proportion of RBBB in 28 studies, Figure S10. Forest plot pooling the proportion of RBBB in subset of 6932 patients with post-TAVI PPI, Figure S11. Forest plot pooling the proportion of RBBB in subset of 39,670 patients without post-TAVI PPI, Figure S12. Baujat plot: impact of the studies on overall heterogeneity in studies reporting on RBBB status, Figure S13. Bubble plots: influence of age on risk for post-TAVI PPI in patients with RBBB, Figure S14. Bubble plots: influence of LVEF on risk for post-TAVI PPI in patients with RBBB, Figure S15. Bubble plots: influence of the year of the study on risk for post-TAVI PPI in patients with RBBB.
