# Supplementary material for: Impact of Bundle Branch Block on Permanent Pacemaker Implantation after Transcatheter Aortic Valve Implantation: A Meta-Analysis
Source: J Clin Med. 2021 Jun 19;10(12):2719. doi: 10.3390/jcm10122719 (PMC8235153; doi:10.3390/jcm10122719)

Supplementary Materials

Figure S1. Flowsheet of the included studies

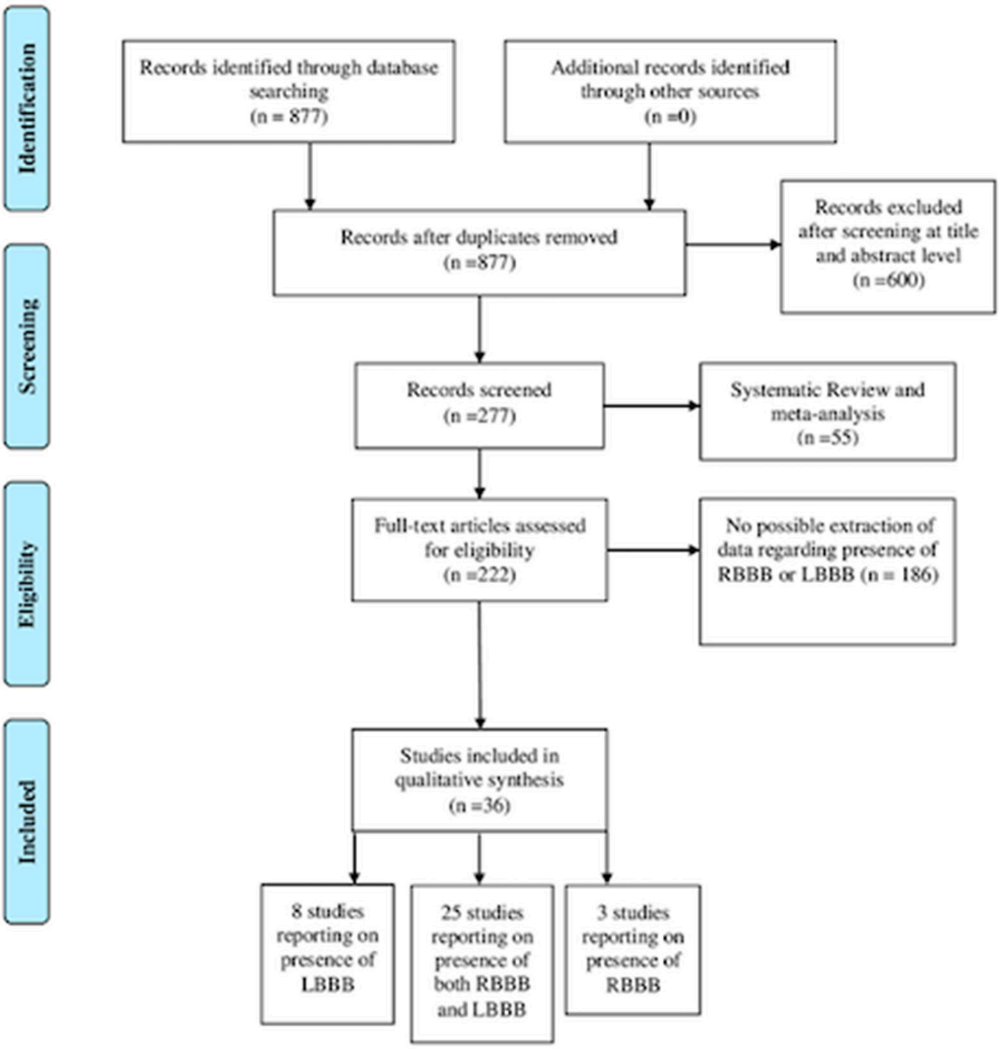

Figure S2. Forest plot pooling the proportion of LBBB in 33 studies.

Study	Events	Total	Weight	IV, Random, 95% CI
1	37	275	2.9%	0.135 [0.097; 0.181]
2	224	818	3.2%	0.274 [0.244; 0.306]
3	50	353	3.0%	0.142 [0.107; 0.182]
4	197	634	3.2%	0.311 [0.275; 0.348]
5	128	668	3.1%	0.192 [0.162; 0.224]
6	121	1151	3.2%	0.105 [0.088; 0.124]
7	106	833	3.1%	0.127 [0.105; 0.152]
8	174	1973	3.2%	0.088 [0.076; 0.102]
9	32	302	2.8%	0.106 [0.074; 0.146]
10	14	250	2.4%	0.056 [0.031; 0.092]
11	74	383	3.0%	0.193 [0.155; 0.236]
12	93	670	3.1%	0.139 [0.114; 0.167]
13	50	578	3.0%	0.087 [0.065; 0.112]
14	39	383	2.9%	0.102 [0.073; 0.137]
15	179	1629	3.2%	0.110 [0.095; 0.126]
16	74	606	3.1%	0.122 [0.097; 0.151]
17	51	672	3.0%	0.076 [0.057; 0.099]
18	724	8148	3.3%	0.089 [0.083; 0.095]
19	398	3404	3.2%	0.117 [0.106; 0.128]
20	107	1025	3.1%	0.104 [0.086; 0.125]
21	61	611	3.0%	0.100 [0.077; 0.126]
22	41	709	2.9%	0.058 [0.042; 0.078]
23	25	283	2.7%	0.088 [0.058; 0.128]
24	1670	18400	3.3%	0.091 [0.087; 0.095]
25	212	1020	3.2%	0.208 [0.183; 0.234]
26	99	1116	3.1%	0.089 [0.073; 0.107]
27	97	386	3.1%	0.251 [0.209; 0.298]
28	41	266	2.9%	0.154 [0.113; 0.203]
29	56	704	3.0%	0.080 [0.061; 0.102]
30	179	1179	3.2%	0.152 [0.132; 0.174]
31	19	256	2.6%	0.074 [0.045; 0.113]
32	52	424	3.0%	0.123 [0.093; 0.158]
33	79	917	3.1%	0.086 [0.069; 0.106]
**Total (95% CI)**		**51026**	**100.0%**	**0.120 [0.104; 0.138]**

Heterogeneity: $Tau^2 = 0.2038$; $Chi^2 = 882.13$, df = 32 ($P < 0.01$); $I^2 = 96\%$

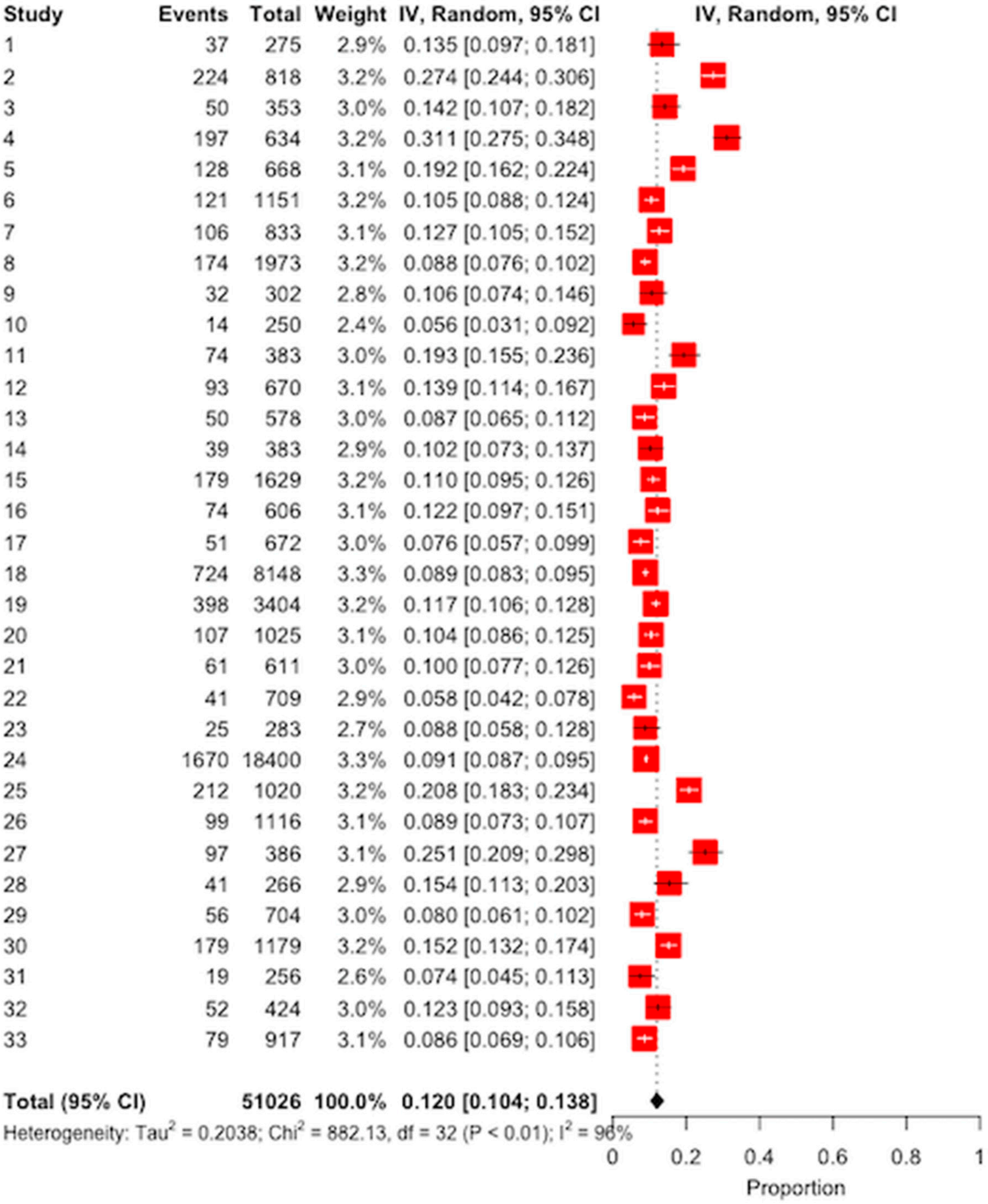

Figure S3. Forest plot pooling the proportion of LBBB in subset of 7 315 patients with post-TAVI PPI.

Study	Events	Total	Weight	IV, Random, 95% CI
1	9	66	3.1%	0.136 [0.064; 0.243]
2	41	142	3.9%	0.289 [0.216; 0.371]
3	10	89	3.2%	0.112 [0.055; 0.197]
4	28	69	3.6%	0.406 [0.289; 0.531]
5	11	29	3.0%	0.379 [0.207; 0.577]
6	12	42	3.2%	0.286 [0.157; 0.446]
7	24	252	3.8%	0.095 [0.062; 0.138]
8	12	173	3.4%	0.069 [0.036; 0.118]
9	5	68	2.6%	0.074 [0.024; 0.163]
10	1	81	1.0%	0.012 [0.000; 0.067]
11	6	52	2.7%	0.115 [0.044; 0.234]
12	15	135	3.5%	0.111 [0.064; 0.177]
13	3	54	2.0%	0.056 [0.012; 0.154]
14	3	44	2.0%	0.068 [0.014; 0.187]
15	37	322	3.9%	0.115 [0.082; 0.155]
16	11	76	3.2%	0.145 [0.075; 0.244]
17	4	146	2.4%	0.027 [0.008; 0.069]
18	253	1949	4.3%	0.130 [0.115; 0.146]
19	84	529	4.1%	0.159 [0.129; 0.193]
20	17	176	3.6%	0.097 [0.057; 0.150]
21	5	54	2.5%	0.093 [0.031; 0.203]
22	4	115	2.4%	0.035 [0.010; 0.087]
23	4	28	2.2%	0.143 [0.040; 0.327]
24	390	1825	4.3%	0.214 [0.195; 0.233]
25	29	73	3.7%	0.397 [0.285; 0.519]
26	8	145	3.0%	0.055 [0.024; 0.106]
27	12	26	2.9%	0.462 [0.266; 0.666]
28	3	35	2.0%	0.086 [0.018; 0.231]
29	20	245	3.7%	0.082 [0.051; 0.123]
30	9	57	3.0%	0.158 [0.075; 0.279]
31	3	38	2.0%	0.079 [0.017; 0.214]
32	6	90	2.8%	0.067 [0.025; 0.139]
33	9	90	3.1%	0.100 [0.047; 0.181]
**Total (95% CI)**		**7315**	**100.0%**	**0.130 [0.106; 0.158]**

Heterogeneity: $Tau^2 = 0.3175$; $Chi^2 = 259.53$, df = 32 ($P < 0.01$); $I^2 = 88\%$

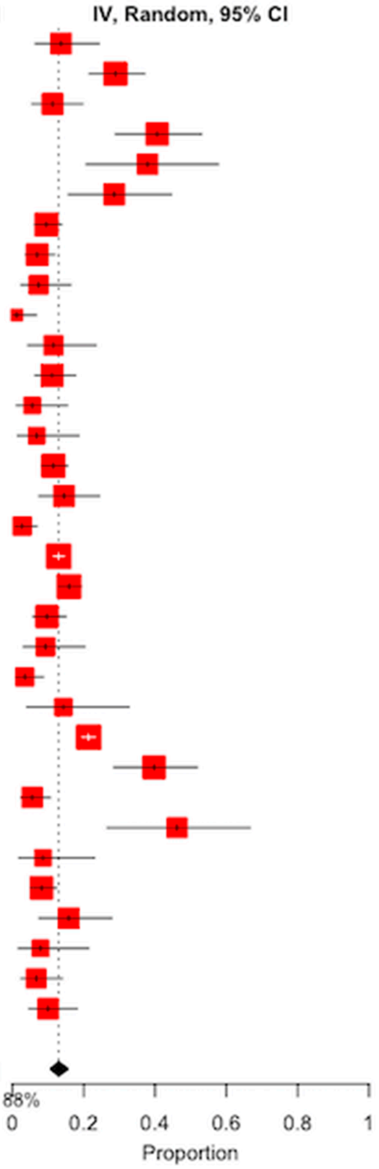

Figure S4. Forest plot pooling the proportion of LBBB in subset of 43 650 patients without post-TAVI PPI.

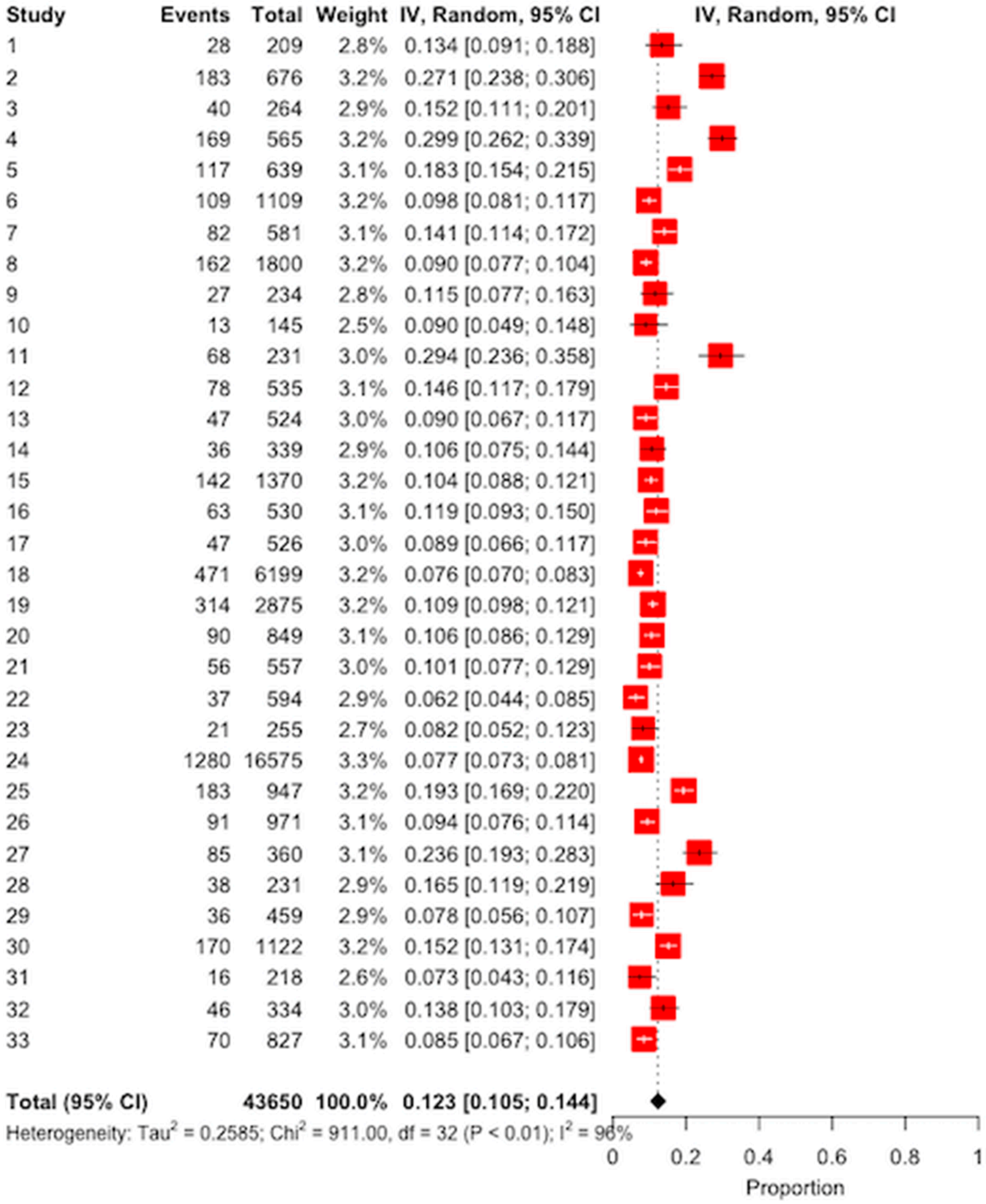

Figure S5. Baujat plot: impact of the studies on overall heterogeneity in studies reporting on LBBB status.

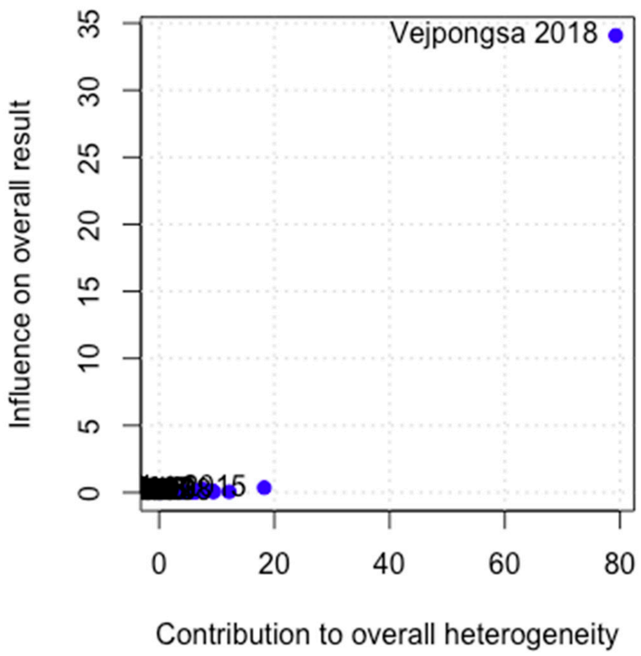

Figure S6. Bubble plots: influence of age on risk for post-TAVI PPI in patients with LBBB.

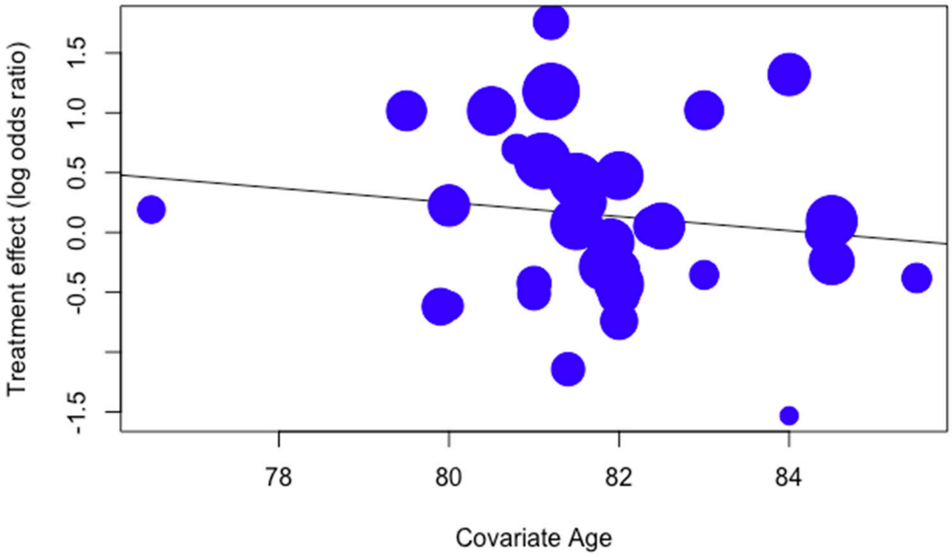

Figure S7. Bubble plots: influence of LVEF on risk for post-TAVI PPI in patients with LBBB

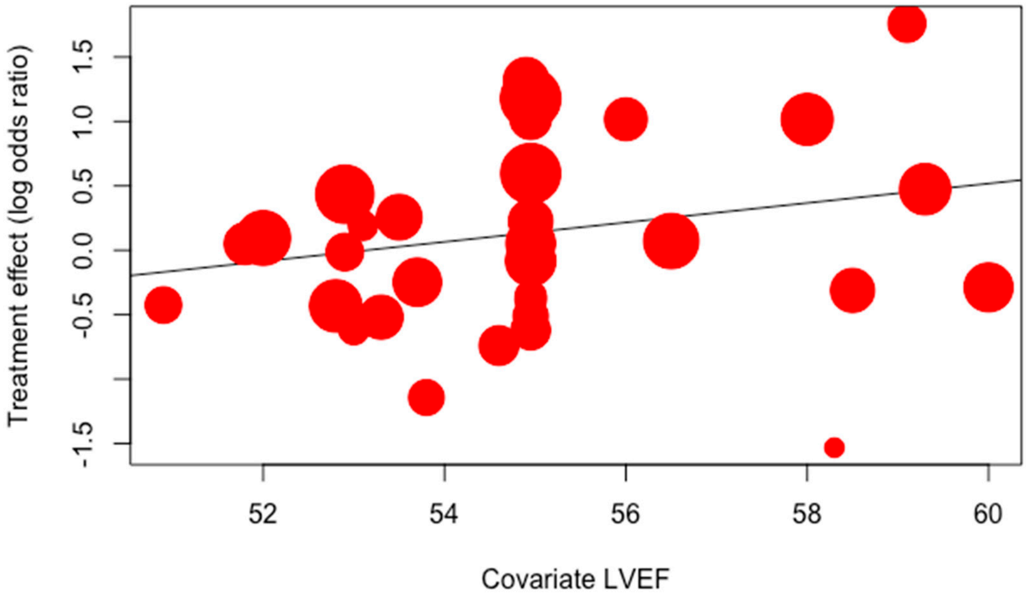

Figure S8. Bubble plots: influence of the year of the study on risk for post-TAVI PPI in patients with LBBB.

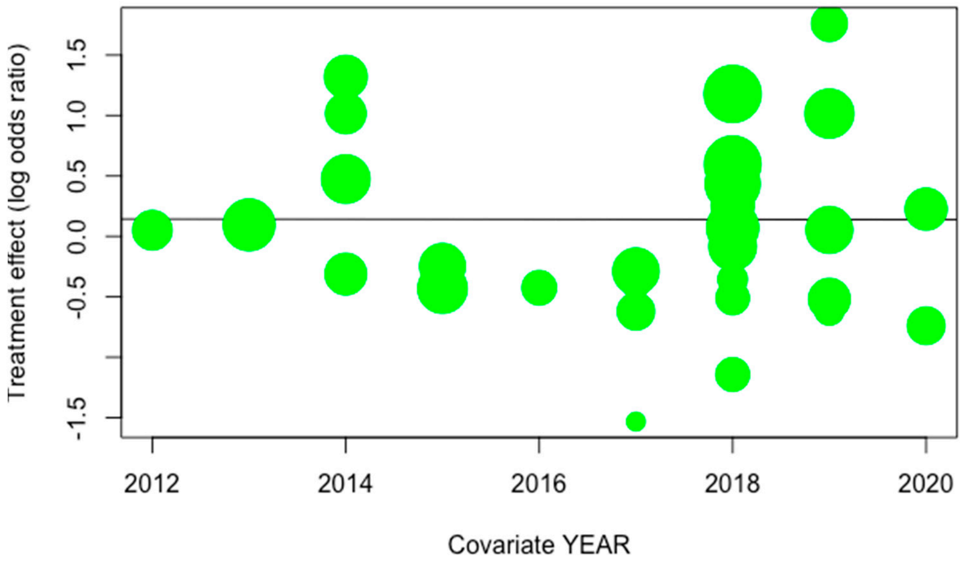

Figure S9. Forest plot pooling the proportion of RBBB in 28 studies.

Study	Events	Total	Weight	IV, Random, 95% CI
1	32	275	3.5%	0.116 [0.081; 0.160]
2	41	353	3.5%	0.116 [0.085; 0.154]
3	115	833	3.7%	0.138 [0.115; 0.163]
4	312	1973	3.7%	0.158 [0.142; 0.175]
5	28	302	3.5%	0.093 [0.062; 0.131]
6	108	749	3.7%	0.144 [0.120; 0.171]
7	22	383	3.4%	0.057 [0.036; 0.086]
8	65	578	3.6%	0.112 [0.088; 0.141]
9	26	250	3.4%	0.104 [0.069; 0.149]
10	71	670	3.6%	0.106 [0.084; 0.132]
11	362	3527	3.7%	0.103 [0.093; 0.113]
12	22	283	3.4%	0.078 [0.049; 0.115]
13	20	606	3.4%	0.033 [0.020; 0.051]
14	37	611	3.5%	0.061 [0.043; 0.083]
15	50	383	3.6%	0.131 [0.098; 0.168]
16	63	709	3.6%	0.089 [0.069; 0.112]
17	98	1025	3.7%	0.096 [0.078; 0.115]
18	113	672	3.7%	0.168 [0.141; 0.199]
19	169	1629	3.7%	0.104 [0.089; 0.120]
20	220	8148	3.7%	0.027 [0.024; 0.031]
21	715	18400	3.7%	0.039 [0.036; 0.042]
22	14	386	3.2%	0.036 [0.020; 0.060]
23	29	266	3.5%	0.109 [0.074; 0.153]
24	101	659	3.7%	0.153 [0.127; 0.183]
25	92	1116	3.7%	0.082 [0.067; 0.100]
26	85	704	3.6%	0.121 [0.098; 0.147]
27	20	256	3.4%	0.078 [0.048; 0.118]
28	130	917	3.7%	0.142 [0.120; 0.166]
**Total (95% CI)**		**46663**	**100.0%**	**0.092 [0.073; 0.116]**

Heterogeneity: $Tau^2 = 0.4576$; $Chi^2 = 1206.83$, df = 27 ($P < 0.01$); $I^2 = 98\%$

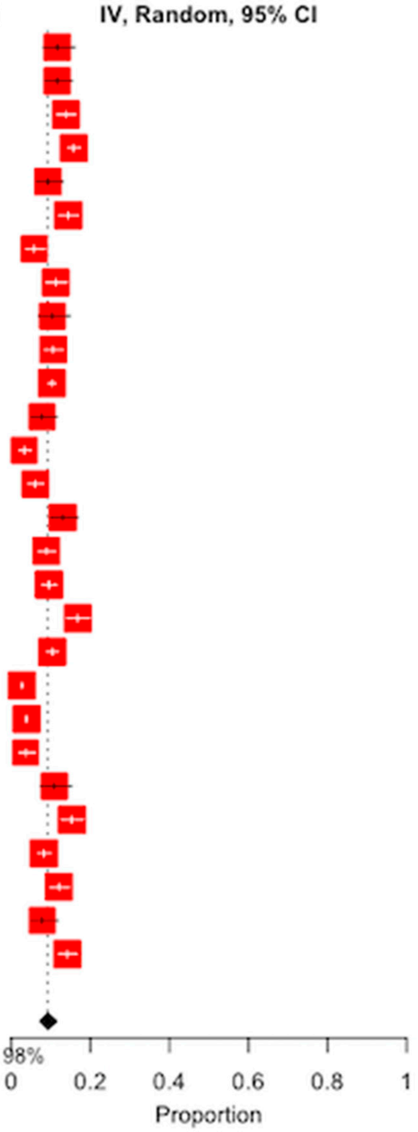

Figure S10. Forest plot pooling the proportion of RBBB in subset of 6 932 patients with post-TAVI PPI

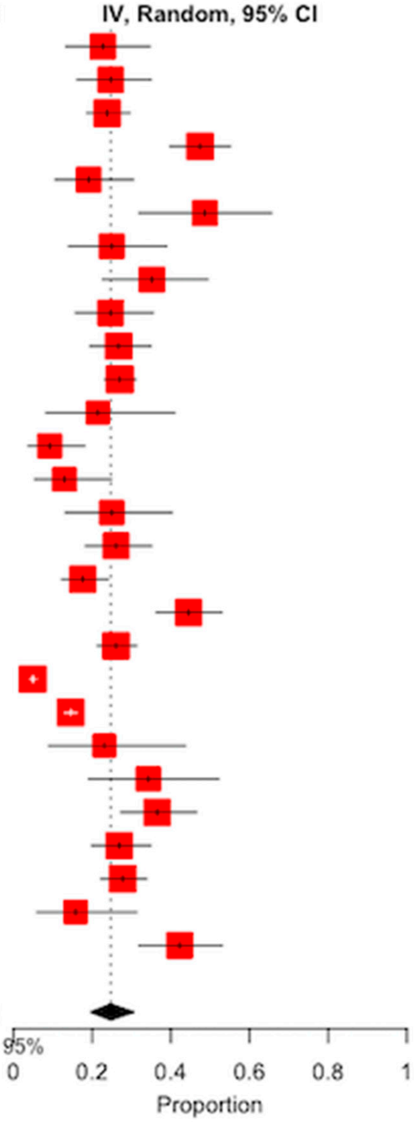

Study	Events	Total	Weight	IV, Random, 95% CI
1	15	66	3.5%	0.227 [0.133; 0.347]
2	22	89	3.6%	0.247 [0.162; 0.350]
3	60	252	3.9%	0.238 [0.187; 0.296]
4	82	173	3.9%	0.474 [0.398; 0.551]
5	13	68	3.4%	0.191 [0.106; 0.305]
6	18	37	3.4%	0.486 [0.319; 0.656]
7	13	52	3.4%	0.250 [0.140; 0.389]
8	19	54	3.5%	0.352 [0.227; 0.494]
9	20	81	3.6%	0.247 [0.158; 0.355]
10	36	135	3.8%	0.267 [0.194; 0.350]
11	137	507	4.0%	0.270 [0.232; 0.311]
12	6	28	2.9%	0.214 [0.083; 0.410]
13	7	76	3.2%	0.092 [0.038; 0.181]
14	7	54	3.1%	0.130 [0.054; 0.249]
15	11	44	3.3%	0.250 [0.132; 0.403]
16	30	115	3.7%	0.261 [0.183; 0.351]
17	31	176	3.8%	0.176 [0.123; 0.241]
18	65	146	3.8%	0.445 [0.363; 0.530]
19	84	322	3.9%	0.261 [0.214; 0.312]
20	96	1949	3.9%	0.049 [0.040; 0.060]
21	265	1825	4.0%	0.145 [0.129; 0.162]
22	6	26	2.9%	0.231 [0.090; 0.436]
23	12	35	3.3%	0.343 [0.191; 0.522]
24	38	104	3.8%	0.365 [0.273; 0.466]
25	39	145	3.8%	0.269 [0.199; 0.349]
26	68	245	3.9%	0.278 [0.222; 0.338]
27	6	38	3.0%	0.158 [0.060; 0.313]
28	38	90	3.7%	0.422 [0.319; 0.531]
**Total (95% CI)**		**6932**	**100.0%**	**0.247 [0.196; 0.306]**

Heterogeneity: $Tau^2 = 0.5682$; $Chi^2 = 503.08$, df = 27 ($P < 0.01$); $I^2 = 95\%$

Figure S11. Forest plot pooling the proportion of RBBB in subset of 39 670 patients without post-TAVI PPI

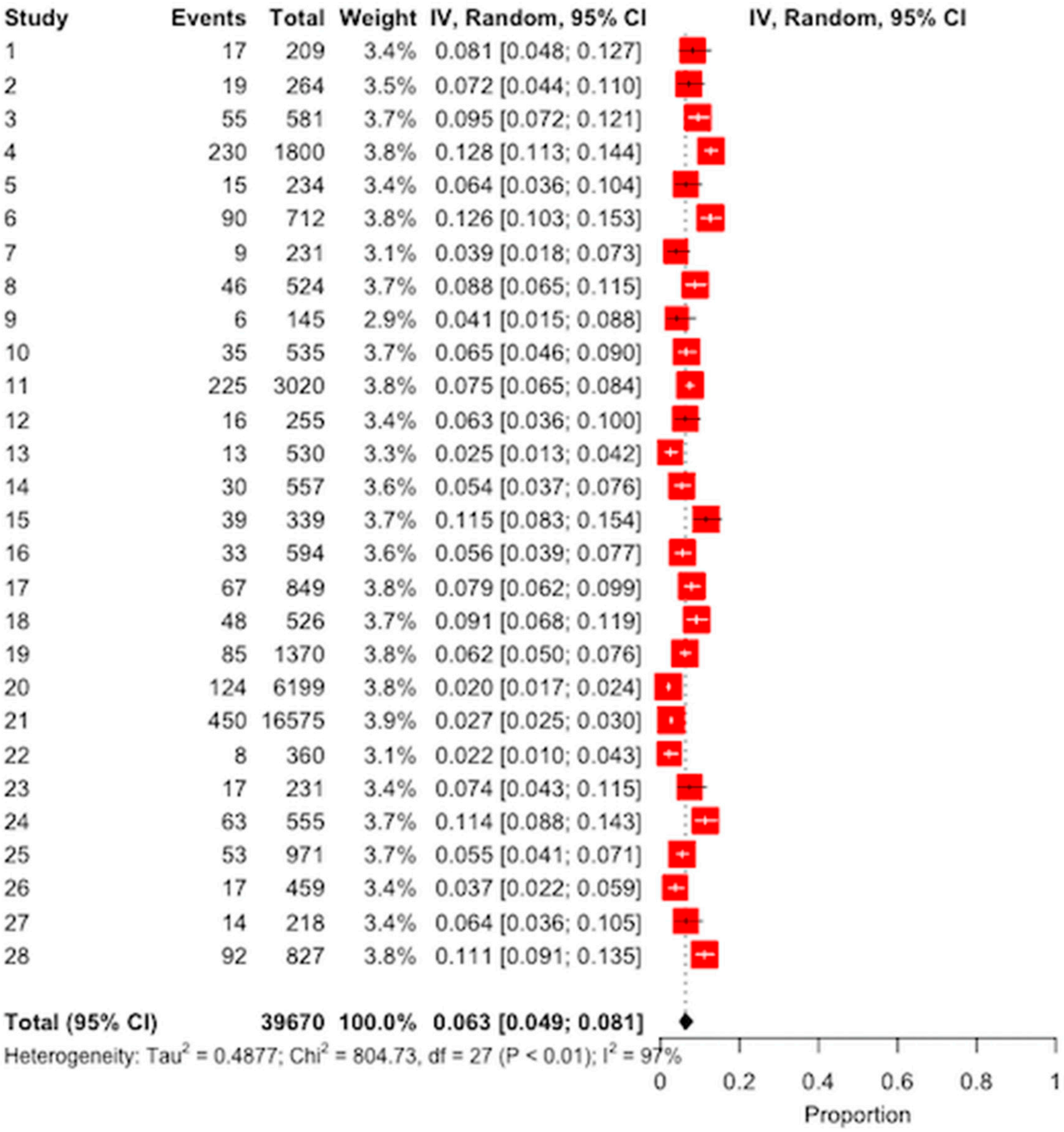

Figure S12. Baujat plot: impact of the studies on overall heterogeneity in studies reporting on RBBB status.

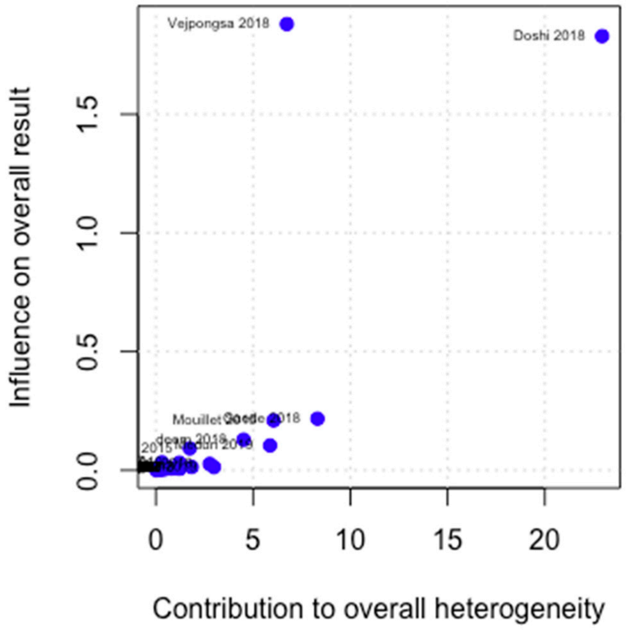

Figure S13. Bubble plots: influence of age on risk for post-TAVI PPI in patients with RBBB.

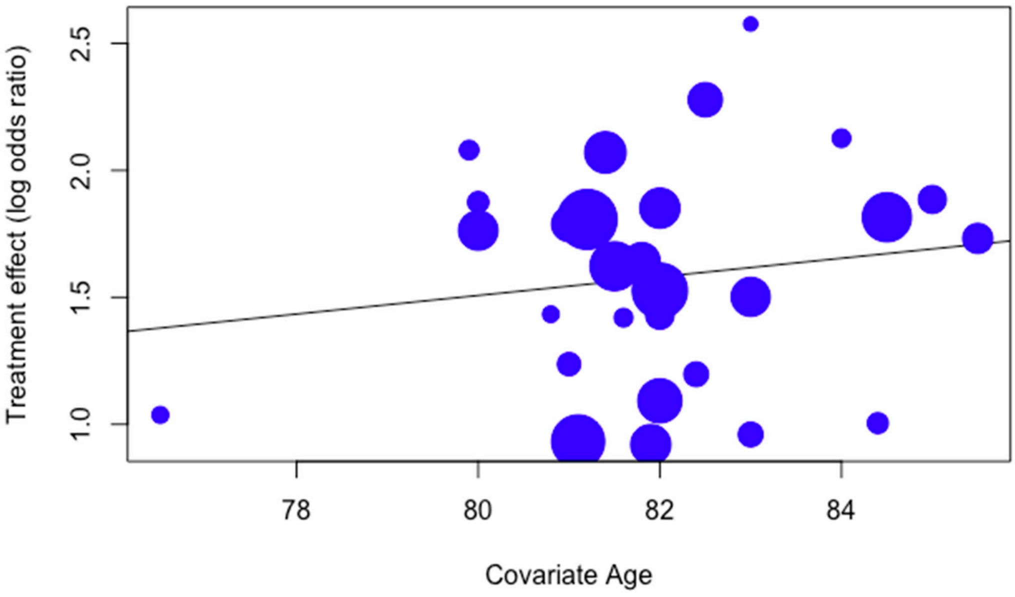

Figure S14. Bubble plots: influence of LVEF on risk for post-TAVI PPI in patients with RBBB

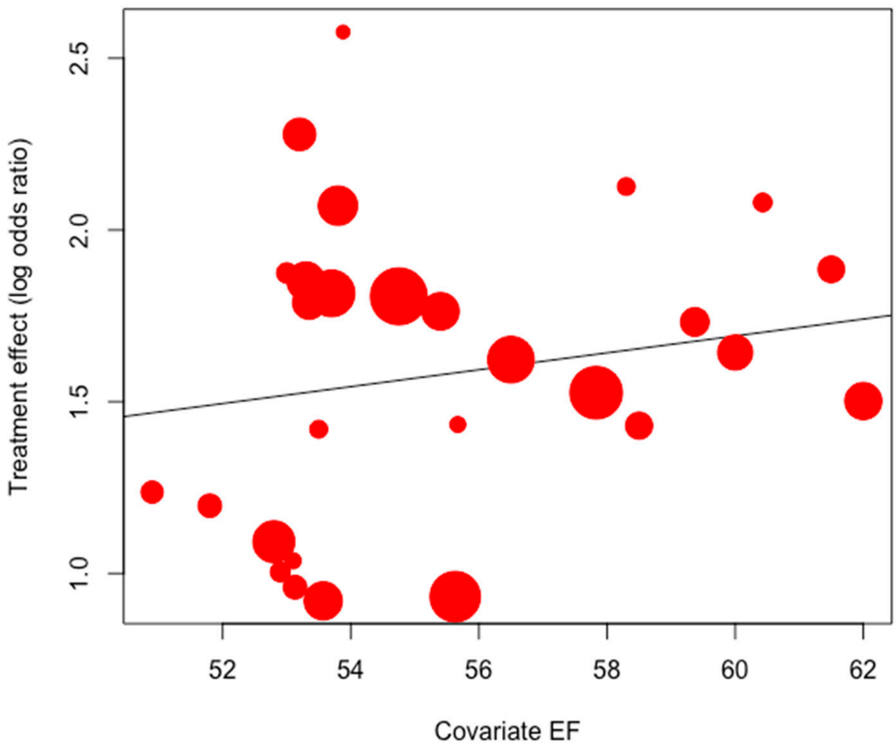

Figure S15. Bubble plots: influence of the year of the study on risk for post-TAVI PPI in patients with RBBB.

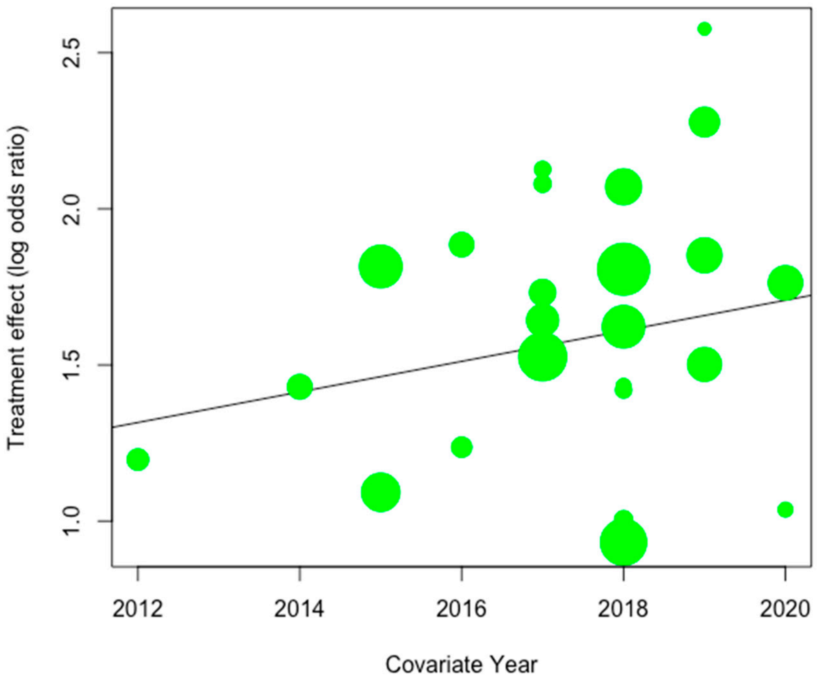

Supplement: Supplementary file 1 [file jcm-10-02719-s001.zip › jcm-1227940-SI.pdf]
